# EfficientNet-CBAM-prototype: an attention-guided EfficientNet framework with dynamic prototype representation for tomato disease classification

**DOI:** 10.3389/fpls.2026.1918175

**Published:** 2026-09-10

**Authors:** E Jansi, Kavitha BR

**Affiliations:** School of Computer Science Engineering and Information Systems (SCORE), Vellore Institute of Technology, Vellore, India

**Keywords:** CBAM, deep learning, dynamic prototype memory, EfficientNet, plant disease classification, prototype network, tomato leaf disease, transfer learning

## Abstract

**Introduction:**

In the field of precision agriculture, one of the major hurdles is the early and accurate identification of plant diseases. Farmers may face serious irreversible loss in yield if there is a delay in diagnosis by even a few days. The CNN model has helped in improving the classification of plant diseases but faces difficulty in distinguishing fine-grained symptoms, has limited generalizability, and is not easily interpretable. Though the deep CNN model performs well in classifying plant diseases, the current methods have several flaws. Most importantly, existing algorithms misclassify visually complex samples due to their limited spatial differentiation of disease-specific morphological features, including lesion borders and necrotic regions. Classification reliability is further compromised by low inter-class embedding separability for visually comparable illness phenotypes. Apart from these representational problems, training instability is still a major problem for attention-based models. Combining randomly initialized attention modules with pretrained backbone networks causes this instability, which still limits practical application.

**Methods:**

We suggest EfficientNet-CBAM-Prototype (ECP-Net), a unique end-to-end deep learning architecture, to overcome these constraints. Three complementary techniques are combined into a single framework by ECP-Net. First, parameter-efficient multi-scale feature extraction is done using an EfficientNetB0 backbone. Second, joint channel-wise and spatial feature recalibration is performed using a stabilized convolutional block attention module (CBAM). Third, a dynamic prototype memory layer uses cosine similarity-based categorization and exponential moving average (EMA) updates to maintain class-representative embedding vectors. To overcome the instability caused by randomly initialized attention weights, we further propose a two-phase training strategy wherein CBAM is frozen during phase 1 to allow prototype stabilization and then jointly fine-tuned with the learning rate in phase 2.

**Results:**

Evaluated on the PlantVillage tomato subset comprising 10 disease classes across a class-balanced split of 10,000 training, 500 validation, and 500 test samples, ECP-Net achieves 98.6% test accuracy, 98.59% F1-score, and 98.65% precision with only 4.80M parameters and 85.04 ms average inference time. These results outperformed baselines including VGG16 (97.00%), ResNet50 (81.20%), MobileNetV2 (81.20%), and CNN (70.00%).

**Discussion:**

Generalization is further validated on 35 real-field tomato leaf images captured under natural, uncontrolled conditions, confirming practical deployment potential.

## 1 Introduction

Agriculture accounts for approximately 4% of global GDP and is the primary livelihood for over 2.5 billion people worldwide. Plant diseases cause annual crop losses estimated between 20–40% of global production. Tomato, one of the most economically important vegetables, is particularly vulnerable to a diverse spectrum of bacterial, fungal, and viral pathogens (Mohanty et al., 2016; Ahmed et al., 2023; Savary et al., 2019). Timely and accurate disease identification is essential for effective intervention, yet traditional diagnostic methods relying on manual expert inspection are inherently limited by scalability, subjectivity, and the shortage of trained agronomists in developing agricultural regions (Jelali, 2024; Shafik et al., 2023).

The rapid advancement of deep learning, particularly convolutional neural networks (CNNs), has opened transformative avenues for automated plant disease recognition from RGB leaf imagery. Pretrained architectures such as VGG16 (Simonyan and Zisserman, 2014), ResNet50 (He et al., 2016), and EfficientNetB0 (Tan and Le, 2019) have demonstrated strong generalization through ImageNet-based transfer learning. However, their direct application to fine-grained plant disease classification presents three fundamental challenges (Borhani et al., 2022; Alzubaidi et al., 2021). First, disease-discriminative features including lesion shape, texture gradients, and spatial distribution of necrotic regions are highly localized and may be suppressed during global pooling operations. Second, a number of tomato disease classes have visual traits that overlap (for example, concentric ring patterns are shared by Early Blight and Target Spot), necessitating geometrically designed embedding spaces that guarantee inter-class separability. Third, the capacity of the traditional softmax classifier to retain consistent class representations across training batches is limited since it does not explicitly specify embedding geometry.

The convolutional block attention module (CBAM) (Woo et al., 2018; Alirezazadeh et al., 2023; Papanastasiou et al., 2023), in particular, has consistently demonstrated gains in focusing network attention on task-relevant spatial regions and feature channels. Concurrently, prototype-based learning (Snell et al., 2017), which is grounded in few-shot learning theory, offers a geometrically interpretable classification framework by comparing query embeddings to class-representative prototype vectors. Despite the independent promise of these approaches, their integration into a unified architecture with a principled training strategy remains largely unexplored in the plant disease classification literature. Such a strategy must specifically address the instability arising from randomly initialized attention modules interacting with pretrained features. Furthermore, even when high benchmark accuracy is achieved, deep learning models frequently fail to generalize to real-world field conditions characterized by variable lighting, occlusion, and background clutter. This underscores the need for deployment-level validation beyond controlled datasets. In this paper, we address this gap with the following principal contributions:

- We proposed ECP-Net, a novel architecture that integrates EfficientNetB0, a stabilized CBAM attention block (incorporating three targeted stability fixes: ReLU pre-activation, residual connections, and post-CBAM batch normalization), and a dynamic prototype memory layer with EMA-based class updates.
- We introduced a two-phase training strategy that decouples prototype stabilization (phase 1: CBAM frozen, LR = 1 × 10^−4^, 15 epochs) from attention fine-tuning (phase 2: full unfreeze, LR = 1 × 10^−5^, up to 20 epochs with early stopping), preventing catastrophic interference between randomly initialized attention weights and pretrained EfficientNet features.
- We formalized the mathematical framework underlying CBAM attention refinement, EMA prototype updates, cosine similarity classification, and the two-phase optimization objective.
- The effectiveness of the proposed network is proved by thorough experimentation on the PlantVillage dataset of tomatoes, where 98.6% and 98.59% F1-score and test accuracy were achieved. Along with the ablation study, the contribution of each architectural component is validated through t-SNE embedding and Grad-CAM visualization.
- The usability of the model in practical agricultural applications was demonstrated through independent evaluation on 35 images of field-grown tomato leaves.

## 2 Related work

### 2.1 Deep learning for plant disease detection

Convolutional neural networks have emerged as the dominant paradigm for image-based plant disease classification, largely driven by the seminal work of Mohanty et al. (2016). Their study demonstrated over 99% accuracy on the controlled PlantVillage benchmark using AlexNet and GoogLeNet, establishing deep learning as a viable foundation for automated crop disease recognition. Ferentinos (2018) reported 99.53% accuracy on PlantVillage using deep CNN architectures, reinforcing the effectiveness of convolutional models for disease recognition. Too et al. (2019) conducted a systematic comparison of DenseNet, InceptionV4, and ResNet on the same benchmark, with DenseNet yielding the strongest performance. Building on this trajectory, Chen et al. (2020a) extended the scope toward practical deployment by applying mobile architectures suited to edge computing environments. EfficientNet (Tan and Le, 2019) has become a parameter-efficient alternative that achieves competitive accuracy at a significantly lower computing cost. This is largely thanks to its compound scaling technique, which simultaneously raises network depth, width, and resolution with a fixed coefficient. More modern works have extended these baselines with transformer architectures (Borhani et al., 2022; Han et al., 2023), lightweight networks (Guan et al., 2023; Thakur et al., 2023), and hybrid CNN-attention designs (Islam et al., 2024; Das et al., 2026). More recently, Rahaman et al. (2026) proposed a customized MobileNetV3Large-based framework achieving 99.37% accuracy across 26 disease classes spanning five crops on a hybrid dataset, while Maurya et al. (2025) introduced RAI-Net, a residual-attention-inception network built on a fine-tuned ResNet18 backbone with channel attention, specifically targeting tomato leaf disease classification and reporting 97.88% accuracy. Udoy et al. (2025) employed a CNN architecture with a tri-attention mechanism for joint classification of potato and tomato blight leaves, achieving 93.98% accuracy. However, none of these works explicitly enforce geometric structure in the embedding space or incorporate attention mechanisms with a principled training strategy. Beyond architectural limitations, a recurring methodological concern in the plant disease classification literature is the exclusive reliance on controlled benchmark datasets such as PlantVillage for evaluation. Several studies have noted that models trained on such datasets exhibit significant performance degradation when applied to field-collected images due to domain shift, a critical barrier to real-world deployment that architectural improvements alone cannot address. The present work addresses both gaps by proposing a novel attention-prototype architecture with a stabilized training strategy and validating generalization on a held-out real-field test set independent of the training distribution.

### 2.2 Attention mechanisms in CNN

In addition to filtering out the irrelevant background, the attention mechanisms will selectively amplify informative parts. Hu et al. (2018) introduced the squeeze and excitation network (SENet), which utilized global average pooling to perform the channel-wise feature recalibration. With the integration of an extra spatial attention branch for generating a 2D attention map concentrating on discriminatory spatial locations, Woo et al. (2018) further developed it as CBAM. The combination of the two mentioned modules will lead to the generation of a joint feature map through channel and spatial attention. Within the agriculture domain, Mi et al. (2020) embedded CBAM within a DenseNet architecture for wheat stripe rust severity grading, and Chen et al. (2020b) used channel attention for detecting rice diseases with a consistent improvement compared to the baseline models without attention (Alirezazadeh et al., 2023; Papanastasiou et al., 2023). Moreover, the attention-driven techniques have been validated not only for general plant pathology standards (Shoaib et al., 2023) but also for tomato leaf disease detection (Sunil and Jaidhar, 2023; Das et al., 2026). The problem of random weight initialization in CBAMs is frequently overlooked. The gradient instability that may arise due to the optimization of these parameters along with the pre-trained backbone features in the first epoch may change the learned representations. However, this problem is mitigated by the proposed approach which involves a two-stage training method combined with a stable CBAM configuration.

### 2.3 Prototype networks and metric learning

Prototype networks were introduced by Snell et al. (2017) for few-shot classification, where each class is represented by the mean embedding of its support examples and classification proceeds via nearest prototype assignment in Euclidean space. A subsequent work by Yang et al. (2018) adapted prototype learning to standard supervised settings, demonstrating improved inter-class separability through convolutional prototype learning. Dynamic prototype representations updated online during training via exponential moving averages have been explored in continual learning contexts (De Lange et al., 2021; Wang et al., 2022; Tarvainen and Valpola, 2017) to maintain stable class representations under non-stationary data distributions. Our proposed prototype layer extends this idea to the supervised fine-grained classification setting using cosine similarity rather than Euclidean distance for scale-invariant prototype matching and combining it with CBAM-refined feature maps for the first time in plant disease classification (Wertheimer et al., 2021; De Silva and Brown, 2023).

### 2.4 Transfer learning strategies

Fine-tuning pretrained CNNs for domain-specific tasks is now standard practice, but the optimal strategy for integrating new randomly initialized modules with frozen pretrained layers requires careful design. Progressive unfreezing strategies (Howard and Ruder, 2018; Kaya et al., 2019) that gradually activate deeper layers during training have shown benefits in NLP. Comparable strategies for vision architectures incorporating attention modules have received limited investigation in the literature. The two-phase training strategy introduced in this work represents a principled and task-specific realization of this concept, tailored to the demands of the CBAM-prototype learning setting.

## 3 Methodology

### 3.1 Overview

**Figure 1:**
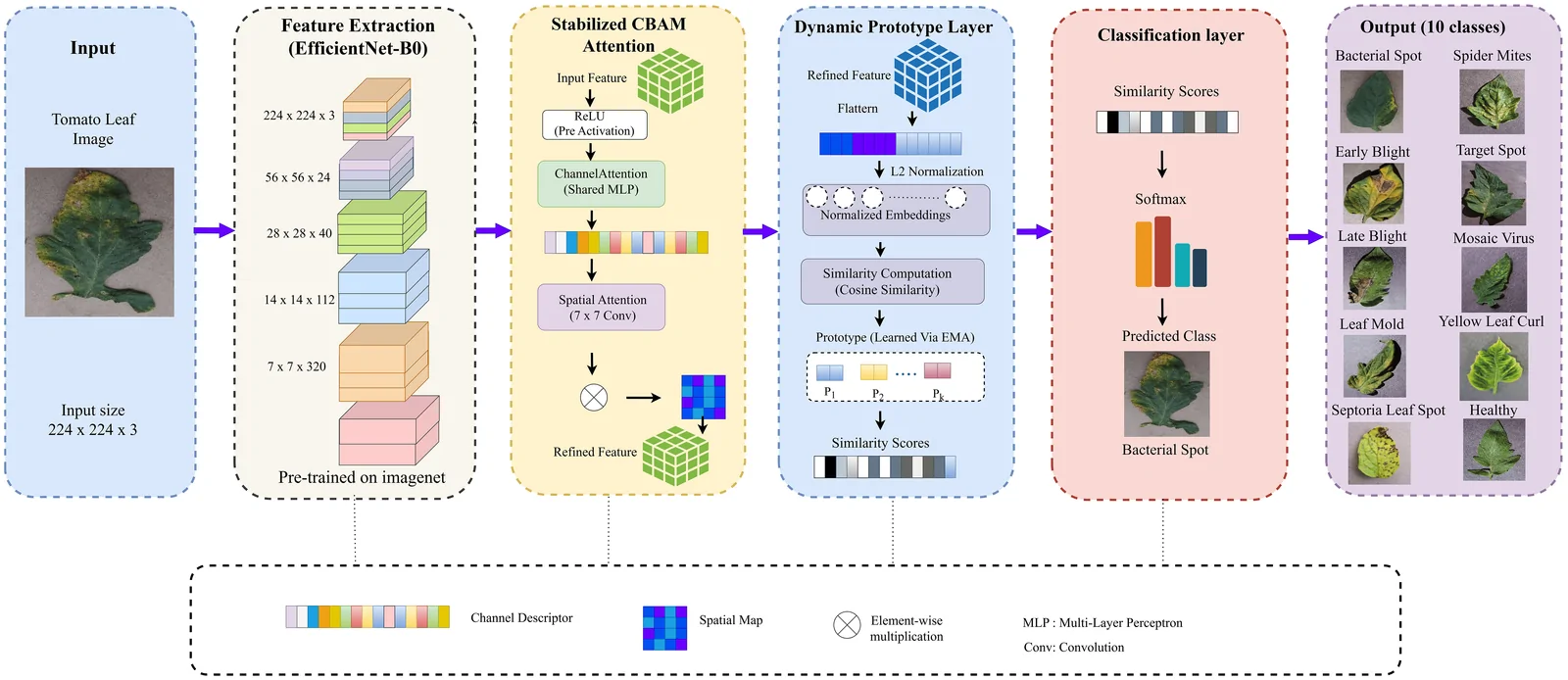
Overall architecture of the proposed ECP-Net framework.

**Figure 1** provides a visual overview of the ECP-Net architecture, illustrating how spatial and channel-wise attention maps are progressively refined before being projected into an embedding space where prototype-based cosine similarity matching drives the final prediction. **Table 1** complements this overview by detailing the layer-wise configuration and corresponding feature map dimensions at each stage of the pipeline, from the EfficientNetB0 backbone through the channel and spatial attention modules to the final prototype-based classification head.

**Table 1:** Layer-wise configuration and feature map dimensions of the proposed ECP-Net model.

Stage	Layer	H × W × C
EfficientNetB0	Stem Conv (3 × 3)	112 × 112 × 32
	MBConv1 (3 × 3)	112 × 112 × 16
	MBConv6 (3 × 3)	56 × 56 × 24
	MBConv6 (5 × 5)	28 × 28 × 40
	MBConv6 (3 × 3)	28 × 28 × 80
	MBConv6 (5 × 5)	14 × 14 × 112
	MBConv6 (3 × 3)	14 × 14 × 192
	MBConv6 (5 × 5)	7 × 7 × 320
	Conv 1×1 (head)	7 × 7 × 1,280
Channel attention	Global Avg Pool	1 × 1 × 1,280
	FC1 (1280 → 160)	1 × 1 × 160
	FC2 (160 → 1280)	1 × 1 × 1,280
	Sigmoid	1 × 1 × 1,280
	Channel multiply	7 × 7 × 1,280
Spatial attention	Avg Pool	7 × 7 × 1
	Max Pool	7 × 7 × 1
	Concat	7 × 7 × 2
	Conv2D (7×7, 2 → 1)	7 × 7 × 1
	Sigmoid	7 × 7 × 1
	Spatial multiply	7 × 7 × 1,280
Stabilization	Residual Add	7 × 7 × 1,280
	BatchNorm	7 × 7 × 1,280
	Global Avg Pool	1 × 1 × 1,280
	BatchNorm	1 × 1 × 1,280
Embedding head	Dense (1280 → 256)	1 × 1 × 256
	ReLU	1 × 1 × 256
	Dropout (0.4)	1 × 1 × 256
	Prototype memory (*C* × 256)	Num classes ×256
Prototype head	L2 normalize (embedding)	1 × 1 × 256
	L2 normalize (prototype)	*C* × 256
	Cosine similarity	1 × 1 × *C*
Softmax output	Softmax activation	1 × 1 × *C*
	Final output (class probabilities)	1 × 1 × *C*

The complete forward pass for an input image $x\in {\mathbb{R}}^{H\times W\times 3}$ is formalized as Equation (1):

(1) $$\hat{y}=\text{Softmax}\left(\text{ProtoLayer}\left(\text{Embed}\left(\text{CBAM}\left(\text{EfficientNet}\left(x\right)\right)\right)\right)\right)$$

where each module is described in detail below.

### 3.2 EfficientNetB0 backbone

EfficientNetB0 [Tan and Le (2019)] serves as the feature extraction backbone, pretrained on ImageNet (1.28M images and 1,000 classes). The network employs a compound scaling rule to simultaneously scale depth (*d*), width (*w*), and resolution (*r*) according to Equation (2):

(2) $$d=\alpha^{\phi },\;w=\beta^{\phi },\;r=\gamma^{\phi }$$

subject to Equation (3):

(3) $$\alpha \cdot \beta^{2}\cdot \gamma^{2}\approx 2,\;\alpha \geq 1,\;\beta \geq 1,\;\gamma \geq 1$$

where *ϕ* = 0 for EfficientNetB0, yielding a baseline network with *α* = 1.2, *β* = 1.1, and *γ* = 1.15. For an input image of size 224 × 224 × 3, the network outputs feature maps as mentioned in Equation (4).

(4) $$F\in {\mathbb{R}}^{7\times 7\times 1280}$$

from the top activation layer. Let *θ_E_* denote the parameters of the embedding head and *θ_P_*denote the parameters of the top (final) layers of the EfficientNet backbone that are fine-tuned during phase 1 while the remaining backbone layers stay frozen. The original classification head is removed and replaced by the proposed attention-prototype stack. In phase 1, the CBAM attention module is fully frozen to allow stable prototype formation using reliable backbone features. In phase 2, all components including CBAM are jointly optimized at a reduced learning rate, with *θ_P_* expanded to cover additional unfrozen backbone layers.

### 3.3 Stabilized CBAM attention block

Standard CBAM (Woo et al., 2018) applied directly to pretrained feature maps may suffer from instability due to the interaction between randomly initialized CBAM parameters and pretrained backbone features. Therefore, three stabilization fixes are introduced.

#### 3.3.1 Channel attention module

Given feature maps, as shown in Equation (5):

(5) $$F\in {\mathbb{R}}^{H\times W\times C}$$

from the backbone, a ReLU pre-activation (fix 1) is first applied to suppress negative activations as defined in Equation (6):

(6) $$F_{\text{relu}}=\text{ReLU}\left(F\right)$$

The channel attention map, defined in Equation (7):

(7) $$M_{c}\in {\mathbb{R}}^{1\times 1\times C}$$

is calculated using a shared multi-layer perceptron (MLP) with reduction ratio *r* = 8 after global average pooling and global max-pooling, as given in Equation (8):

(8) $$M_{c}\left(F\right)=\sigma \left(\text{MLP}\left(\text{AvgPool}\left(F_{\text{relu}}\right)\right)+\text{MLP}\left(\text{MaxPool}\left(F_{\text{relu}}\right)\right)\right)$$

The MLP operation is defined in Equation (9):

(9) $$\text{MLP}\left(x\right)=W_{1}\cdot \text{ReLU}\left(W_{0}\cdot x\right)$$

where, as specified in Equation (10):

(10) $$W_{0}\in {\mathbb{R}}^{\frac{C}{r}\times C},\;W_{1}\in {\mathbb{R}}^{C\times \frac{C}{r}}$$

and the sigmoid activation function is represented by *σ*.

The channel-refined feature map is then obtained as shown in Equation (11):

(11) $$F_{\text{ch}}=M_{c}\left(F\right)⊗F_{\text{relu}}$$

where ⊗ denotes element-wise multiplication.

#### 3.3.2 Spatial attention module

The spatial attention map, given in Equation (12):

(12) $$M_{s}\in {\mathbb{R}}^{H\times W\times 1}$$

is generated by concatenating channel-wise average pooling and max pooling outputs followed by a 7 × 7 convolution, as shown in Equation (13):

(13) $$M_{s}\left(F_{\text{ch}}\right)=\sigma \left({\text{Conv}}_{7\times 7}\left(\left[{\text{AvgPool}}_{c}\left(F_{\text{ch}}\right);{\text{MaxPool}}_{c}\left(F_{\text{ch}}\right)\right]\right)\right)$$

where []; denotes channel concatenation.

The spatially refined feature map is computed as shown in Equation (14):

(14) $$F_{\text{sp}}=M_{s}\left(F_{\text{ch}}\right)⊗F_{\text{ch}}$$

#### 3.3.3 Stabilization fixes

Fix 2 introduces a residual connection to preserve backbone feature integrity during early training as mentioned in Equation (15):

(15) $$F_{\text{res}}=F_{\text{sp}}+F_{\text{relu}}$$

Fix 3 applies batch normalization [Ioffe and Szegedy (2015)] to standardize feature distributions before the embedding head as given in Equation (16):

(16) $$F_{\text{cbam}}=\text{BatchNorm}\left(F_{\text{res}}\right)$$

The final CBAM output, shown in Equation (17)

(17) $$F_{\text{cbam}}\in {\mathbb{R}}^{7\times 7\times 1280}$$

maintains spatial dimensions while highlighting areas and channels that are disease-discriminative.

### 3.4 Embedding head

A compact embedding space is created by projecting the CBAM-refined feature maps as shown in Equation (18):

(18) $$z={\text{Dropout}}_{0.4}\left(\text{ReLU}\left(W_{e}\cdot \text{GlobalAvgPool}\left(\text{BatchNorm}\left(F_{\text{cbam}}\right)\right)+b_{e}\right)\right)$$

where $W_{e}\in {\mathbb{R}}^{256\times 1280}$, $b_{e}\in {\mathbb{R}}^{256}$ are learnable parameters.

The resulting embedding vector is: $z\in {\mathbb{R}}^{256}$ which serves as input to the prototype memory layer.

### 3.5 Dynamic prototype memory layer

The prototype memory layer maintains a non-trainable prototype matrix as defined in Equation (19):

(19) $$P\in {\mathbb{R}}^{C\times D}$$

where *C* = 10 represents the number of disease classes and *D* = 256 denotes the embedding dimension. Each row *P_i_*corresponds to the prototype vector of class *i*, initialized using Glorot uniform initialization.

#### 3.5.1 Online EMA prototype update

During training, prototype vectors are updated online using an exponential moving average (EMA) of batch embeddings (Wang et al., 2022; Tarvainen and Valpola, 2017). Given a training batch, as shown in Equation (20)

(20) $$B=\left\{\left(z_{j},y_{j}\right)\right\}$$

the batch mean embedding for class *i* is computed as shown in Equation (21):

(21) $$\mu_{i}^{\left(t\right)}=\frac{1}{\left|B_{i}\right|}\sum_{j\in B_{i}}z_{j}$$

(22) $$B_{i}=\{j:\arg\max(y_{j})=i\}$$

where $\left|{ℬ}_{i}\right|$ is defined in Equation (22) the cardinality (number of samples) of class *i* in the current batch ℬ. The prototype update rule is conditionally applied only when class *i* appears in the batch as shown in Equation (23) and follows Equation (24):

(23) $$P_{i}^{\left(t\right)}=m\cdot P_{i}^{\left(t-1\right)}+\left(1-m\right)\cdot \mu_{i}^{\left(t\right)},\text{ if }\left|B_{i}\right|>0$$

(24) $$P_{i}^{\left(t\right)}=P_{i}^{\left(t-1\right)},\text{ if }\left|B_{i}\right|=0$$

where *m* = 0.9 denotes the momentum coefficient. This update is non-differentiable and executed outside the computational graph, ensuring stable prototype evolution without interfering with backbone gradient propagation.

#### 3.5.2 Cosine similarity classification

At inference time, classification is performed by computing the cosine similarity between the L2-normalized query embedding and all L2-normalized class prototypes, as shown in Equation (25):

(25) $$\text{sim}\left(z,P_{i}\right)=\left(\frac{z}{{\left\|z\right\|}_{2}}\right)\cdot {\left(\frac{P_{i}}{{\left\|P_{i}\right\|}_{2}}\right)}^{T}$$

The complete similarity vector across all classes forms the logit vector as given in Equation (26):

(26) $$\text{logits}=\left[\text{sim}\left(z,P_{0}\right),\text{sim}\left(z,P_{1}\right),\ldots ,\text{sim}\left(z,P_{C-1}\right)\right]\in {\mathbb{R}}^{C}$$

Cosine similarity normalizes the embedding magnitude, thereby improving robustness to scale variations in feature representations. The final class probabilities are obtained using the Softmax function as shown in Equation (27):

(27) $$P(y=i|x)=\frac{\text{exp}\;\left(\frac{\text{sim}\left(z,P_{i}\right)}{\tau }\right)}{\sum_{k}\text{exp}\;\left(\frac{\text{sim}\left(z,P_{k}\right)}{\tau }\right)}$$

### 3.6 Two-phase training strategy

**Figure 2:**
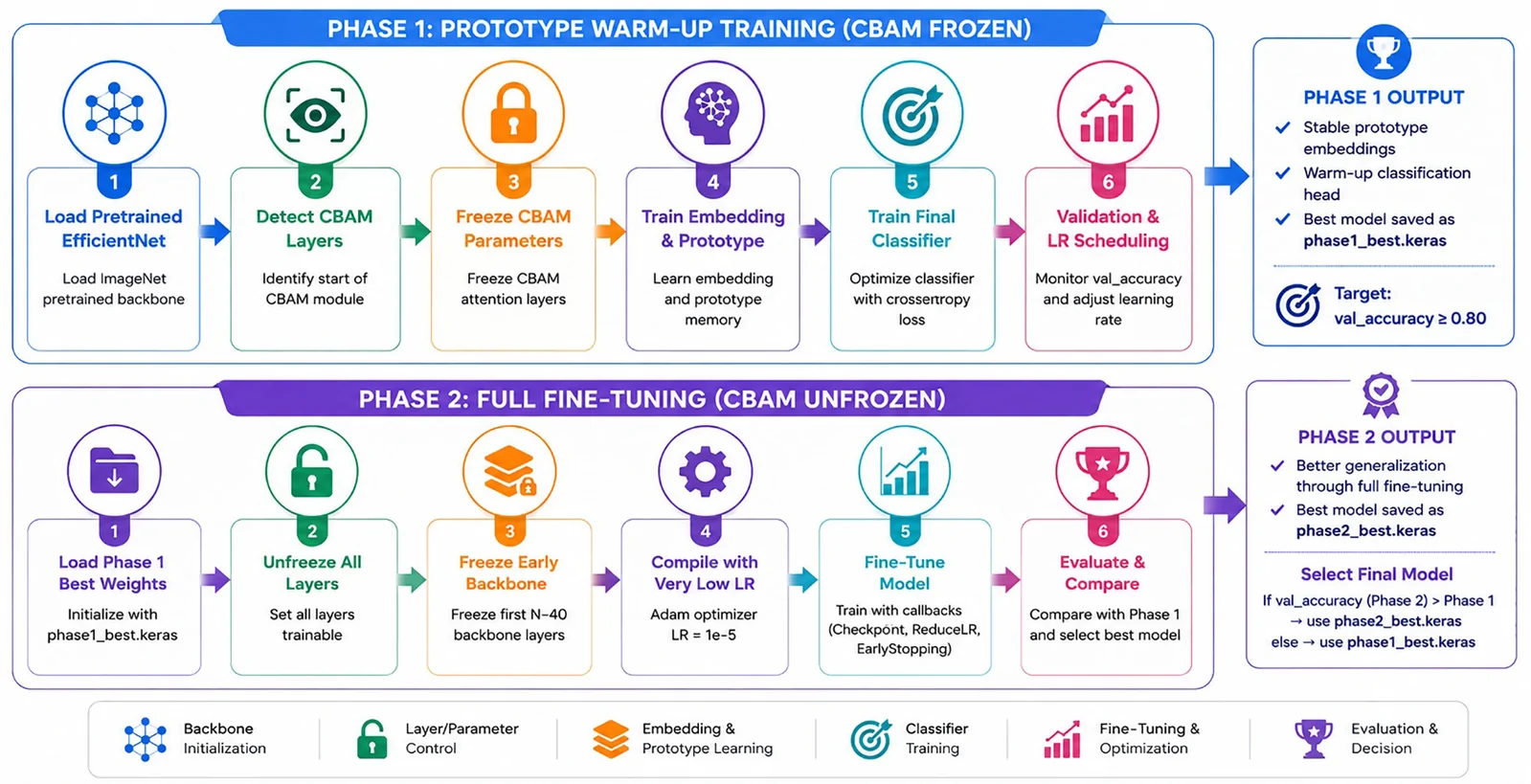
Overview of the two-phase training strategy in ECP-Net, depicting restricted gradient flow during prototype stabilization (phase 1) and full parameter optimization during attention calibration (phase 2).

A central contribution of this work is the proposed two-phase training protocol, which is designed to resolve the incompatibility between randomly initialized CBAM weights and pretrained EfficientNetB0 representations. As illustrated in **Figure 2**, the training process is divided into two sequential phases. In phase 1, the EfficientNetB0 backbone is frozen while the newly introduced CBAM attention module, embedding head, and prototype memory components are trained to learn stable feature representations. In phase 2, selected layers of the backbone are progressively unfrozen and jointly optimized with the newly added modules, enabling effective adaptation of pretrained features to the target tomato disease classification task while preserving the benefits of transfer learning.

#### 3.6.1 Phase 1: prototype stabilization (CBAM frozen)

In phase 1, all CBAM layers are frozen such that Equation (28):

(28) $$\frac{\partial L}{\partial \theta_{\text{CBAM}}}=0$$

The optimization objective is formulated in Equation (29):

(29) $$\underset{\theta_{E},\theta_{P}}{\min}\;L_{\text{CE}}\left(\text{Softmax}\left(\text{ProtoLayer}\left(\text{Embed}\left({\text{CBAM}}_{\text{frozen}}\left(\text{EfficientNet}\left(x\right)\right)\right)\right)\right),y\right)$$

where:

- *θ_E_* denotes the embedding head parameters, and
- *θ_P_* denotes the full trainable EfficientNetB0 backbone parameters.

The frozen-CBAM training pipeline for phase 1 is visually depicted in **Figure 2**, where gradient flow is restricted to the embedding head and the top EfficientNet backbone layers while the CBAM weights remain static.

Training is performed for *T*_1_ = 15 epochs using the Adam optimizer with *β*_1_ = 0.9, *β*_2_ = 0.999, and learning rate *η*_1_ = 1 × 10^−4^. The objective of phase 1 is to establish geometrically stable prototype representations by freezing the randomly initialized CBAM weights, allowing the embedding head and prototype memory to learn from reliable, unperturbed EfficientNetB0 features before attention-based modulation is introduced.

#### 3.6.2 Phase 2: joint fine-tuning (CBAM unfrozen)

In phase 2, the best checkpoint obtained from phase 1 is loaded, and all CBAM layers and embedding head parameters are jointly optimized alongside the full EfficientNetB0 backbone, which has remained trainable since phase 1.

The optimization objective becomes, as shown in Equation (30):

(30) $$\underset{\theta_{\text{CBAM}},\theta_{E},\theta_{P}}{\min}L_{\text{CE}}\left(\text{Softmax}\left(\text{ProtoLayer}\left(\text{Embed}\left(\text{CBAM}\left(\text{EfficientNet}\left(x\right)\right)\right)\right)\right),y\right)$$

Training is continued using a substantially reduced learning rate: *η*_2_ = 1 × 10^−5^, such that: $\eta_{2}=\frac{\eta_{1}}{10}$.

This reduced learning rate prevents destabilization of the prototype representations formed during phase 1. Early stopping is applied with *P* = 6 epochs of patience along with best-weight restoration. Additionally, the ReduceLROnPlateau scheduler reduces the learning rate by a factor of 0.5 if the validation loss fails to improve for four consecutive epochs. By constraining the learning rate in phase 2, the CBAM attention gates learn to calibrate against stable and semantically rich features rather than the noisy activations that dominate early random initialization.

### 3.7 Loss function and optimization

To handle the class imbalance in the PlantVillage tomato dataset, categorical cross-entropy loss is applied with per-class weighting, as shown in Equation (31):

(31) $$L_{\text{CE}}=-\sum_{i=1}^{C}w_{i}\cdot y_{i}\cdot \log\;(P(y=i|x))$$

here *C* is the total number of disease classes, $w_{i}\in {\mathbb{R}}^{+}$ denotes the weight assigned to class *i* to penalize underrepresented categories, $y_{i}\in \left\{0,1\right\}$ is the ground-truth label, and P(y=i|x)∈(0,1) is the predicted probability for class *i* given input *x*

(32) $$w_{i}=\frac{N_{\text{total}}}{C\cdot N_{i}}$$

where:

- *N*_total_ is the total number of training samples, and
- *N_i_*denotes the number of training samples belonging to class *i*, as given in Equation (32).

## 4 Experimental setup

### 4.1 Dataset

**Figure 3:**
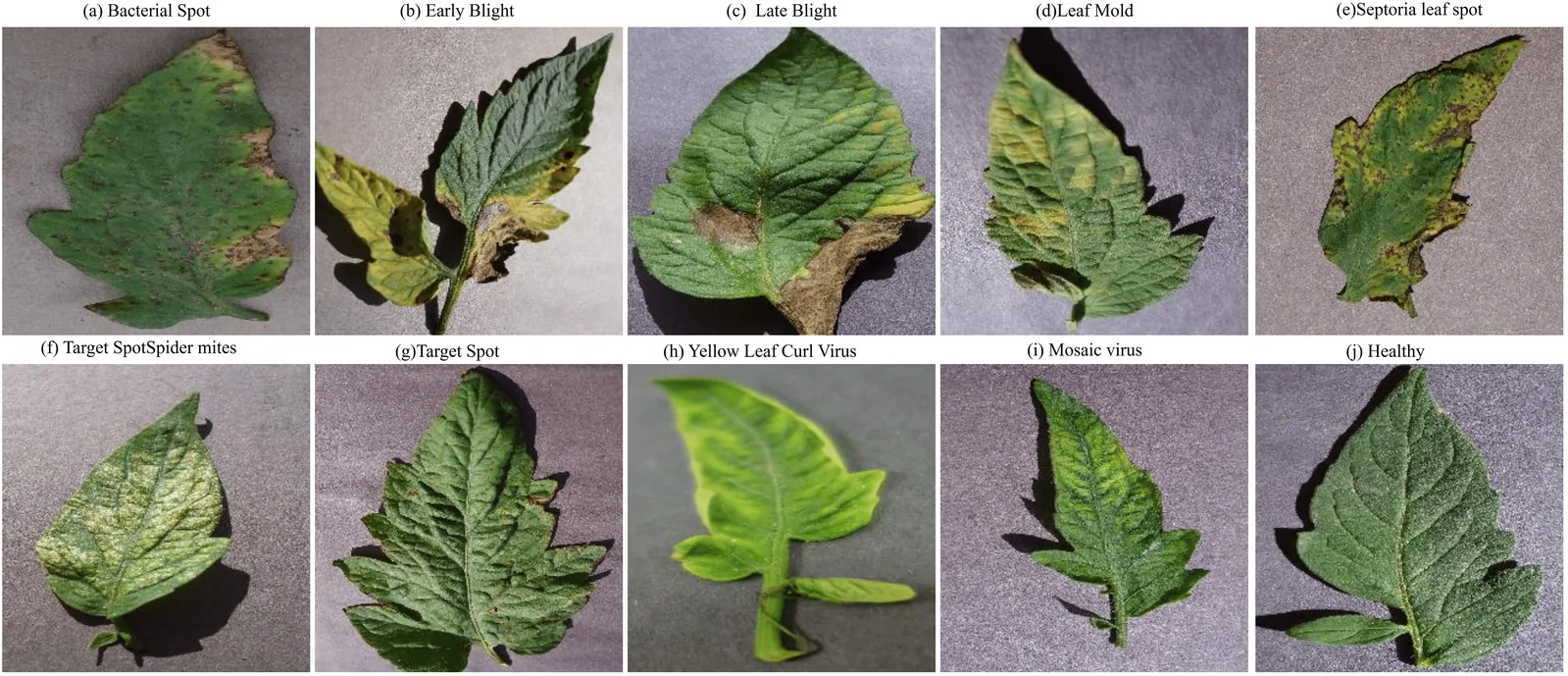
Representative leaf images from the PlantVillage dataset across all 10 tomato disease classes used in this study: **(a)** Bacterial spot, **(b)** Early blight, **(c)** Late blight, **(d)** Leaf mold, **(e)** Septoria leaf spot, **(f)** Target spot and spider mites, **(g)** Target spot, **(h)** Yellow leaf curl virus, **(i)** Mosaic virus, **(j)** Healthy. Images are RGB photographs acquired under controlled laboratory conditions.

**Figure 4:**
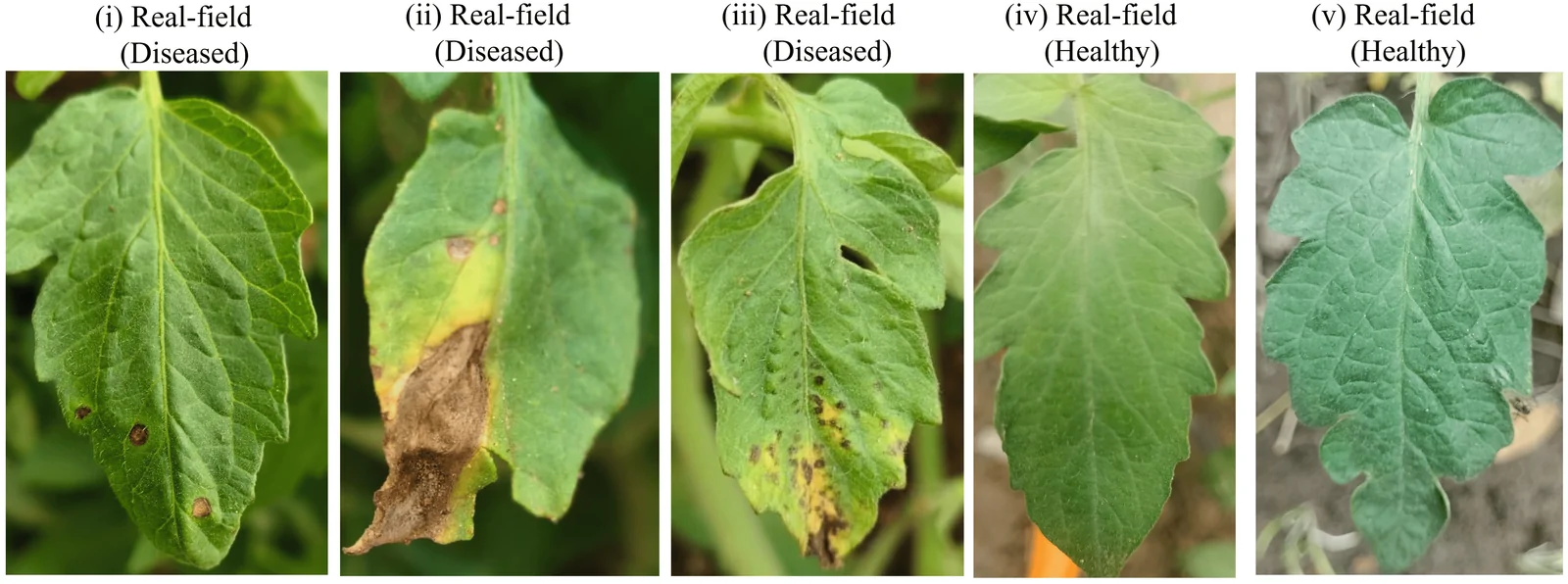
Field-collected tomato leaf images acquired under natural, uncontrolled agricultural conditions exhibiting variations in illumination, background complexity, and disease appearance. Classes shown: healthy and diseased.

All experiments were performed on the PlantVillage dataset (Hughes and Salathe´, 2015), which contains RGB leaf images from 14 crop species covering 38 disease classes; all of the images were acquired under controlled laboratory conditions. The tomato subset is selected from this dataset for evaluation, covering 10 agriculturally relevant categories: bacterial spot, early blight, late blight, leaf mold, *Septoria* leaf spot, spider mites (two-spotted), target spot, tomato mosaic virus, tomato yellow leaf curl virus, and healthy. Representative images for each category are shown in **Figure 3**. All samples are resized to 224 × 224 pixels to satisfy the input dimensionality requirements of the EfficientNetB0 backbone. A class-balanced partitioning strategy was applied to ensure uniform representation across all categories, allocating 1,000 images per class for training and 50 images per class each for validation and testing. This yielded a total of 10,000 training, 500 validation, and 500 test images across the 10 tomato disease classes. Uniform class representation across all partitions mitigates the risk of model bias toward any dominant category during both training and performance evaluation. Online data augmentation is applied exclusively during training to mitigate overfitting and improve model robustness against real-field imaging variability (Harakannanavar et al., 2022; Thakur et al., 2023). The augmentation pipeline comprises random rotation (± 20°), random zoom (± 20%), horizontal flipping, and width/height shift (± 10%), simulating natural variation in leaf orientation, camera distance, and lighting conditions encountered in field-captured images. To assess cross-domain generalization beyond the controlled PlantVillage distribution, a real-field test set was independently compiled comprising 35 tomato leaf images collected from agricultural fields under natural lighting and uncontrolled environmental conditions. Due to the absence of disease-level annotations in the field collection, the images were organized into two categories: diseased (*n* = 25) and healthy (*n* = 10). To support this binary evaluation protocol, a separate class-balanced partition was derived from the PlantVillage tomato subset, wherein the nine disease categories were consolidated into a single diseased class, comprising 900 images per class for training and 100 images for validation, using the same ECPNet architecture without modification. Representative samples from the field collection are shown in **Figure 4**. This real-field test set was maintained as a strictly held-out evaluation partition, with no overlap with the PlantVillage training or validation data, to provide an unbiased assessment of deployment-level generalization.

### 4.2 Baseline models

ECP-Net is compared against five baseline architectures trained under identical data and evaluation conditions as follows:

- Custom CNN: A three-block convolutional architecture trained from scratch, serving as the nonpretrained lower bound
- VGG16 (Simonyan and Zisserman, 2014): A deep architecture containing approximately 138M parameters, included to demonstrate the parameter-efficiency advantage of the proposed model
- ResNet50 (He et al., 2016): Residual network architecture employing skip connections and evaluated under the same transfer learning protocol
- MobileNetV2 (Sandler et al., 2018): A lightweight depthwise-separable architecture designed for mobile deployment
- EfficientNetB0 (Tan and Le, 2019): The backbone component of ECP-Net evaluated independently as an ablation baseline

### 4.3 Implementation details

All models are implemented using TensorFlow 2.x with Keras and executed in a GPU-enabled Kaggle environment. EfficientNetB0 is initialized using ImageNet pretrained weights. The CBAM module uses:

- Reduction ratio: *r* = 8 for the channel attention MLP
- Spatial attention convolution kernel: 7 × 7

Prototype momentum is set as *m* = 0.9 Training uses a batch size of 16.

The Adam optimizer is employed throughout with the following parameters: *β*_1_ = 0.9, *β*_2_ = 0.999, and *ϵ* = 1 × 10^−7^.

## 5 Results and discussion

This section reports the experimental outcomes of the proposed two-phase transfer learning framework applied to tomato leaf disease classification. Performance is assessed through training and validation accuracy curves, corresponding loss trajectories, and a confusion matrix computed on the held-out test set of 50 samples per class across all 10 disease categories.

### 5.1 Training and validation accuracy

**Figure 5:**
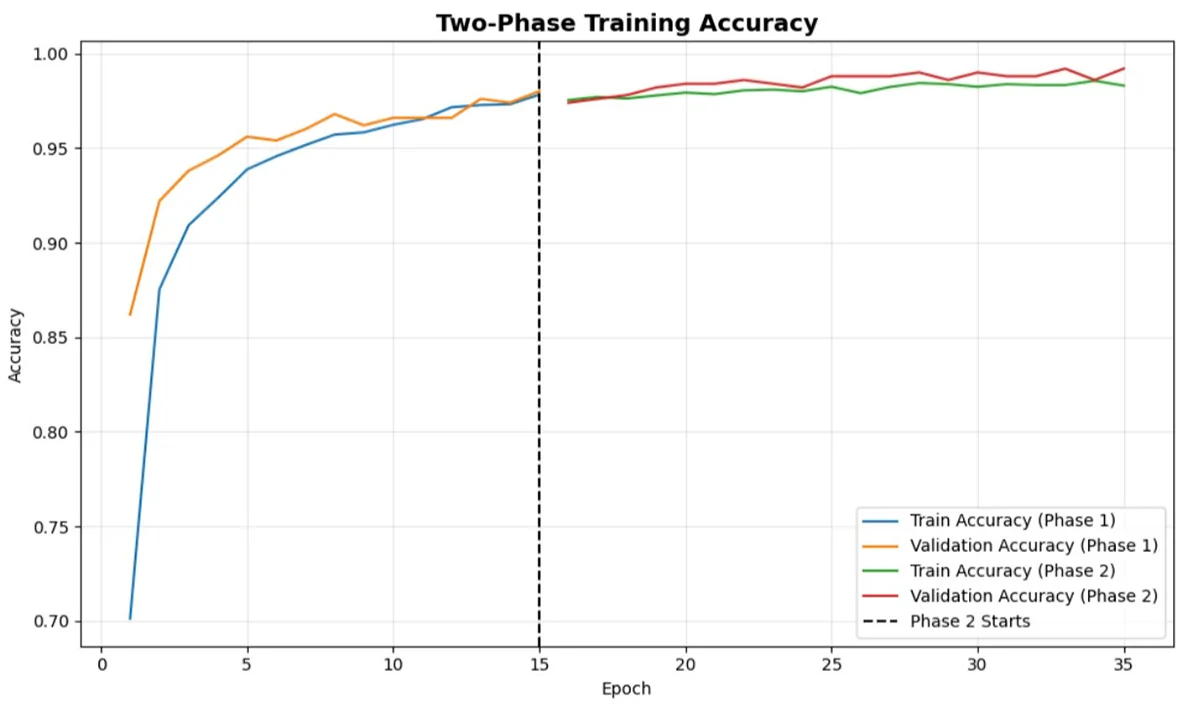
Training and validation accuracy curves across both phases: Phase 1 (epochs 1–15) with frozen backbone layers and phase 2 (epochs 16–35) with selective fine-tuning.

**Figure 5** presents the evolution of training and validation accuracy across both phases of the proposed training protocol. During phase 1 (epochs 1–15), backbone parameters were kept frozen, and only the classifier head underwent optimization. Training accuracy climbed steeply from roughly 0.70 at epoch 1 to 0.98 by epoch 15, a trend attributable to the swift adaptation of the freshly initialized dense layers to the extracted features from the backbone. Interestingly, validation accuracy consistently surpassed training accuracy throughout phase 1, opening at approximately 0.86 and stabilizing near 0.98 by epoch 15. This behavior stems from the application of dropout regularization during training, which intentionally suppresses a fraction of activations, whereas inference is performed without dropout, yielding a higher observed accuracy on the validation set (Srivastava et al., 2014).

In phase 2 (epochs 16–35), full fine-tuning was performed by unfreezing the deeper convolutional layers of the backbone at a substantially reduced learning rate. Throughout phase 2, both training and validation accuracy settled within a tight band of 0.975–0.990, with validation accuracy marginally ahead of training accuracy at most epochs. This tight convergence indicates that the model generalizes well to unseen data without exhibiting overfitting, consistent with prior work employing two-stage fine-tuning strategies on plant disease datasets. A slight upward drift in validation accuracy approaching epoch 35, reaching ≈ 0.990, indicates that sustained fine-tuning at reduced learning rates can produce incremental yet meaningful gains in generalization performance.

### 5.2 Training and validation loss

**Figure 6:**
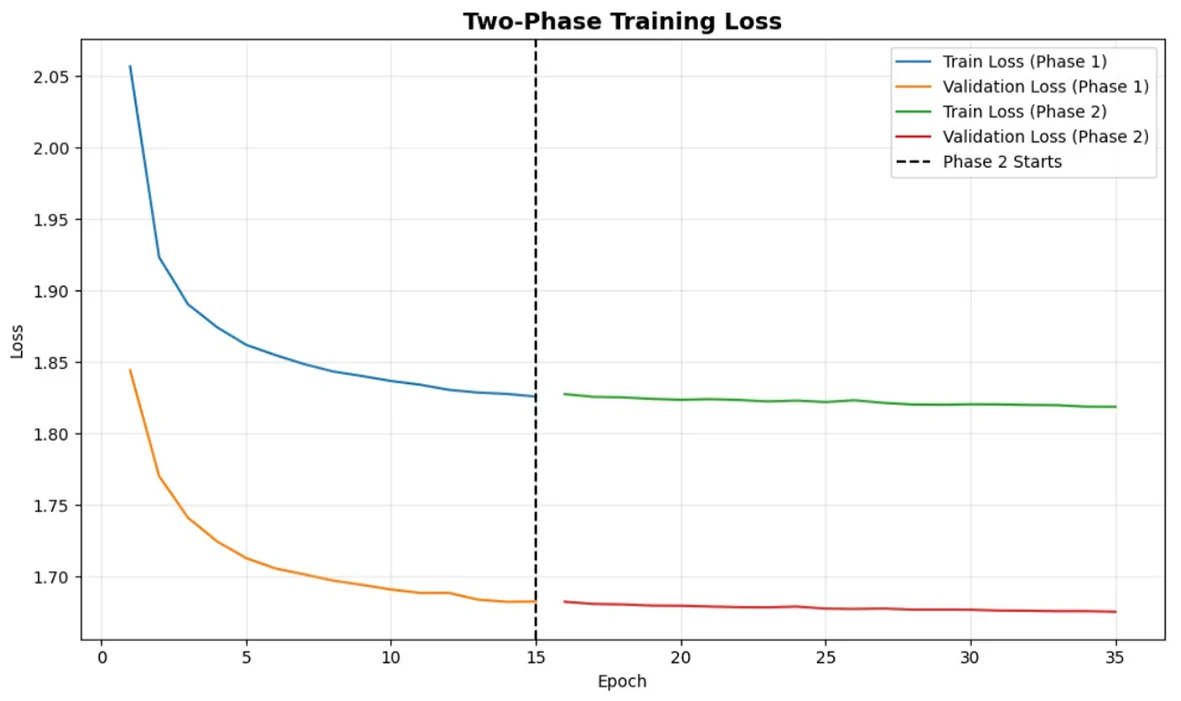
Training and validation loss trajectories over phase 1 (epochs 1–15) and phase 2 (epochs 16–35) of the proposed two-phase training protocol.

**Figure 6** depicts the corresponding loss trajectories across both phases. In phase 1, training loss dropped from 2.05 to roughly 1.83, while validation loss fell more sharply from 1.84 to 1.69, a gap consistent with the suppressive effect of dropout applied exclusively during training. This persistently lower validation loss relative to training loss aligns with the accuracy trends observed in **Figure 5**, together confirming that the model is well regularized throughout training (Goodfellow et al., 2016).

The transition to phase 2 introduced a notable behavioral shift: the training loss (green curve) initialized at 1.83, closely matching the phase 1 terminal value, and gradually declined to approximately 1.82 over 20 additional epochs, demonstrating stable optimization under the reduced learning rate schedule. The validation loss (red curve) began marginally below the training loss at ≈1.69 and exhibited a gradual downward trend, settling near 1.68 by the close of epoch 35. The near-flat profile of both loss curves in phase 2 indicates that the model reached a well-converged optimum, and no signs of divergence or catastrophic forgetting were observed during backbone fine-tuning (Yosinski et al., 2014). It is noted that the absolute loss values (phase 1: 2.05 → 1.83; phase 2: 1.83 → 1.82) appear elevated relative to conventional categorical cross-entropy ranges; this is attributable to the class-weighted loss formulation (“Section 3.7”), wherein per-sample loss contributions are scaled by inverse class-frequency weights, inflating the absolute loss magnitude without any adverse effect on the classification behavior of the model.

### 5.3 Confusion matrix and per-class performance

**Figure 7:**
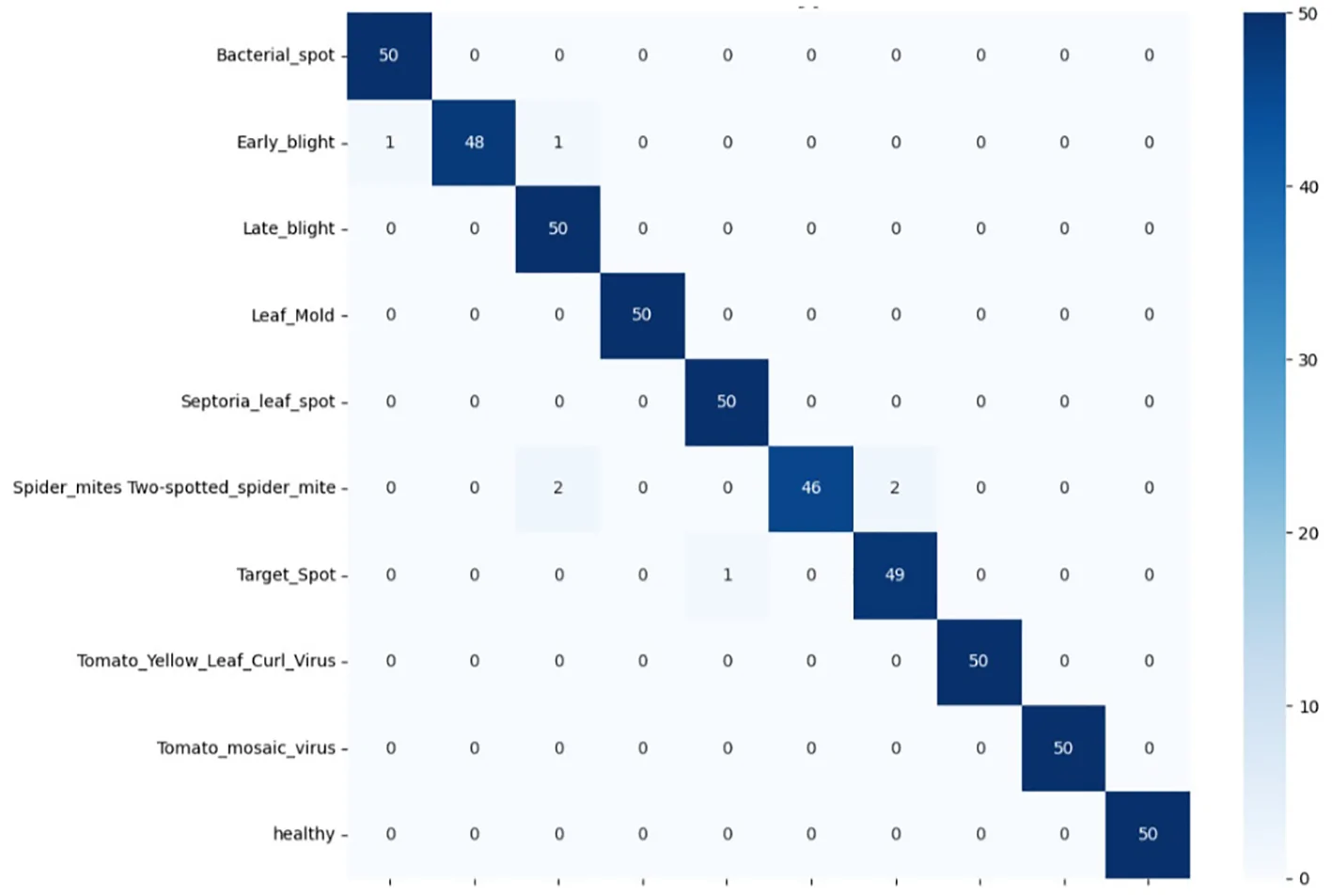
Confusion matrix computed on the held-out test set comprising 50 samples per class across the 10 tomato disease categories.

**Figure 7** presents the confusion matrix evaluated on the test set. Of the 500 total test samples (50 per class), the model correctly classified 493, yielding an overall test accuracy of 98.6%. Seven misclassifications were observed across three classes:

- Early blight was misclassified once as bacterial spot and once as late blight;
- Spider mite, specifically the two-spotted spider mite, was confused twice with late blight and twice with target spot; and
- Target spot produced one false prediction toward Septoria leaf spot.

The remaining seven classes—bacterial spot, late blight, leaf mold, *Septoria* leaf spot, tomato yellow leaf curl virus, tomato mosaic virus, and healthy—achieved perfect precision and recall (50/50 correct), demonstrating the robustness of the model across the majority of disease categories.

The observed confusions are visually intuitive: *early blight* and *late blight* share overlapping brown necrotic lesion morphology, while spider mite damage and *target spot* both manifest as small, dispersed circular lesions on the leaf surface. These inter-class similarities represent an inherent challenge in fine-grained visual classification of plant diseases and suggest that augmenting the dataset with hard-negative samples or applying class-specific attention mechanisms may further reduce misclassification rates.

Beyond these quantitative results, we further examine the spatial reasoning, embedding geometry, and prototype structure of ECP-Net through three complementary qualitative analyses in the following section.

### 5.4 Qualitative analysis and model interpretability

Beyond quantitative performance metrics, a rigorous evaluation of deep learning models for plant disease diagnosis demands qualitative validation of the learned representations. This section presents three complementary analyses: gradient-weighted class activation mapping (Grad-CAM) for spatial interpretability, t-SNE feature embedding for representational separability, and prototype memory visualization for structural analysis of the class-level embedding space. These analyses together provide converging evidence that the proposed model learns biologically meaningful, discriminative, and well-structured representations of tomato leaf diseases.

#### 5.4.1 Gradient-weighted class activation mapping

To assess the spatial reasoning of the proposed model, Grad-CAM (Selvaraju et al., 2020) was applied to the final convolutional layer of the fine-tuned backbone. Grad-CAM computes the gradient of the class score with respect to the feature maps of the target layer, producing a localization heatmap that highlights the image regions most influential in the decision of the model.

**Figure 8:**
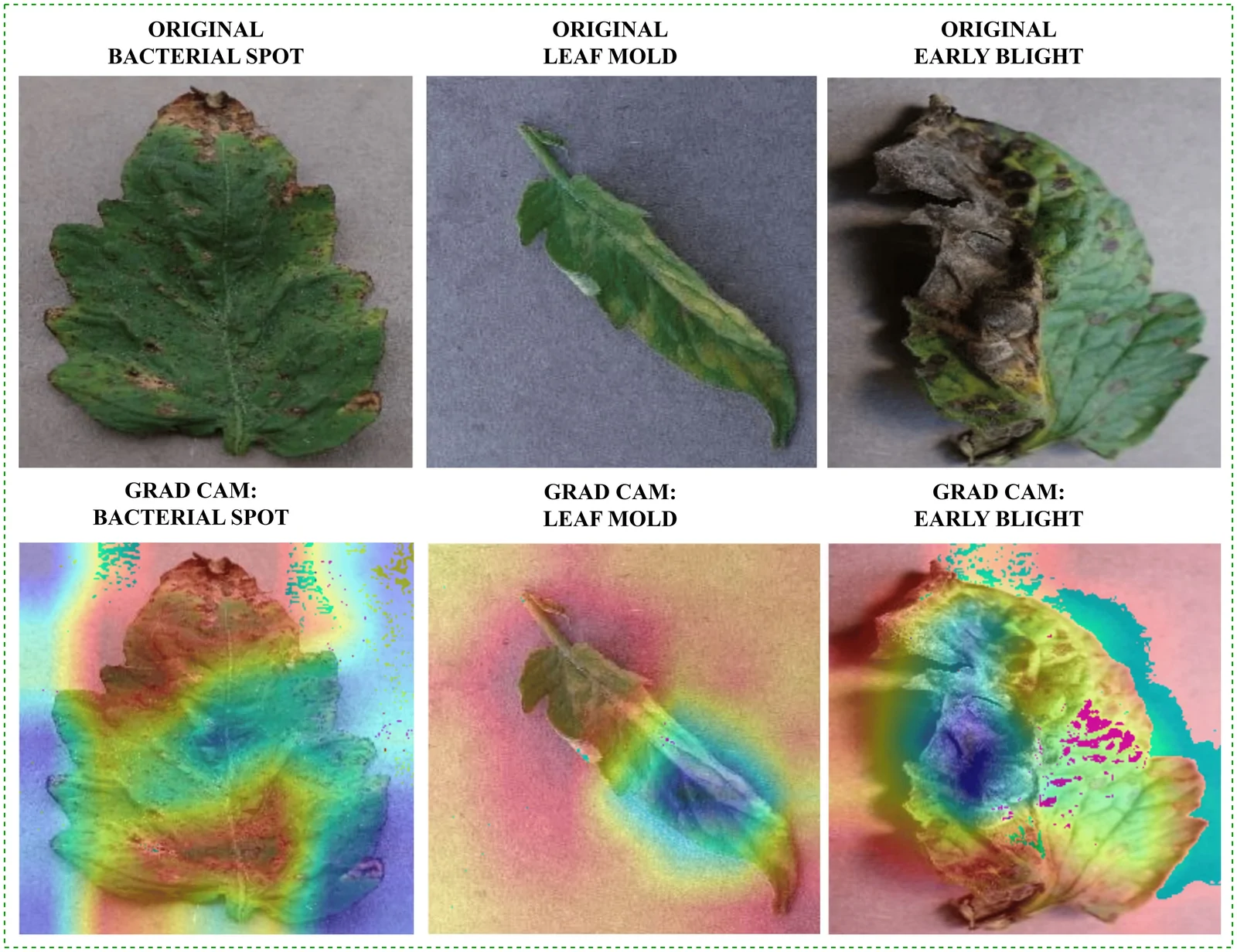
Grad-CAM visualization for a leaf mold test sample. Top: Original input image. Bottom: Grad-CAM saliency overlay with the prediction (leaf mold) of the model superimposed. Warm colors (red/orange) indicate low activation regions, and cool colors (green/blue) indicate high-attention discriminative regions.

**Figure 8** presents the Grad-CAM overlay for a representative leaf mold sample correctly classified by the model. The saliency map reveals that the highest activation (blue/green regions) is concentrated along the central leaf lamina and the mold-affected surface patches, which are the visually diagnostic regions for leaf mold infection characterized by yellow-green chlorotic patches on the upper surface and grayish-white fungal growth on the underside (van der Maaten and Hinton, 2008). Peripheral and background regions, by contrast, register minimal activation (pink/red areas), indicating that the decisions of the model are driven by lesion-relevant features rather than scene context. This absence of background sensitivity confirms that the proposed framework avoids spurious correlation learning, a well-documented failure mode in classifiers trained on dataset-biased benchmarks (Snell et al., 2017).

This finding carries particular weight given that leaf mold attained perfect classification accuracy (50/50) in the confusion matrix, suggesting that the model has genuinely learned disease-discriminative texture patterns rather than dataset artifacts. Grad-CAM visualizations suggest that this stems from the capacity of the model to localize fine-grained fungal texture patterns on the leaf surface rather than relying on coarse shape or color-based cues. These findings align with the growing emphasis on explainable AI (XAI) in precision agriculture, where actionable model outputs require spatial justification (Yosinski et al., 2014).

#### 5.4.2 t-SNE feature embedding visualization

To examine the quality of the learned feature space, t-SNE was applied to reduce the 256-dimensional prototype memory embeddings to two dimensions for all test samples. t-SNE is a non-linear dimensionality reduction technique that preserves local neighborhood structure, making it well suited for visualizing the degree of inter-class separation in high-dimensional embedding spaces.

**Figure 9:**
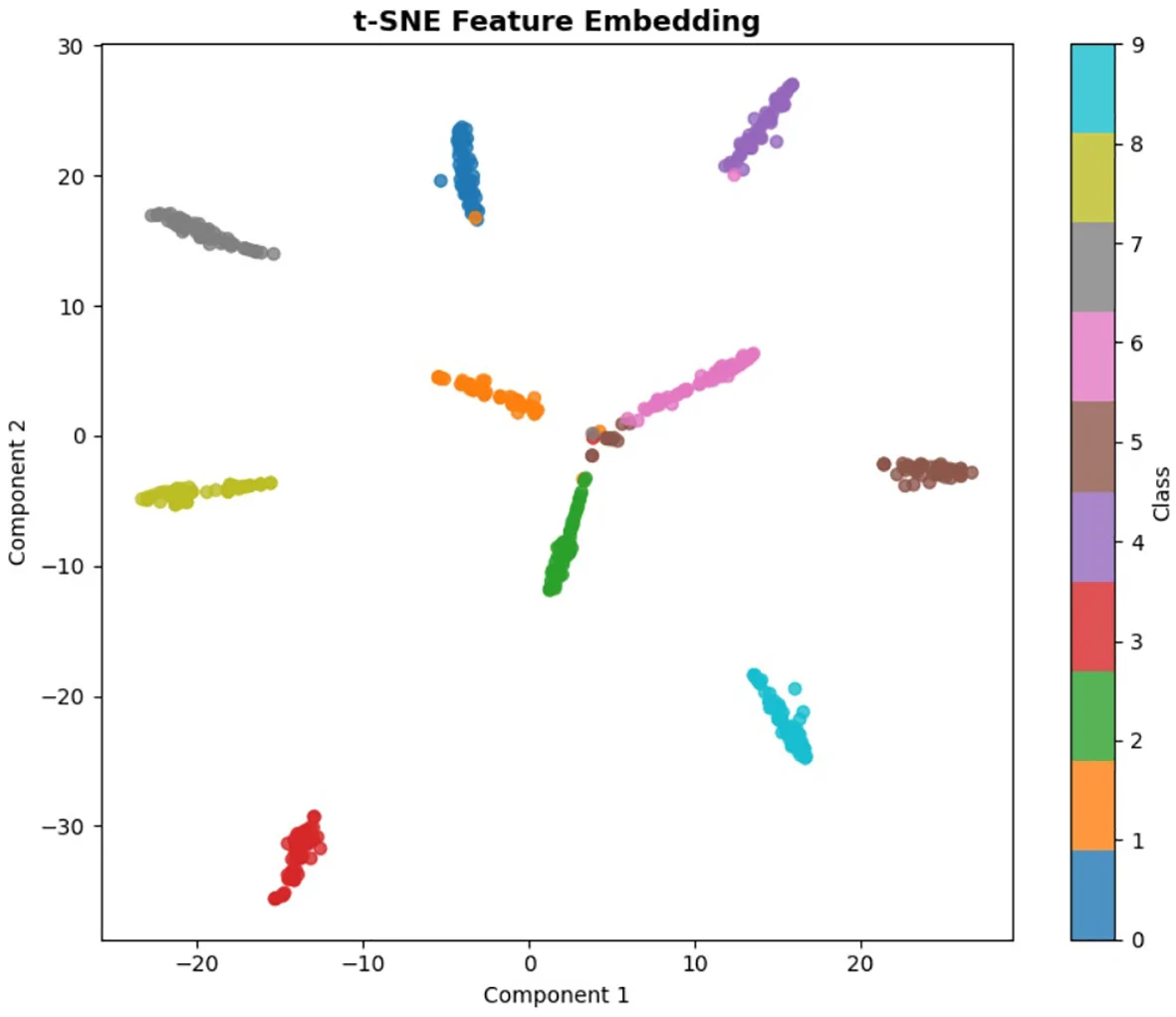
t-SNE projection of the 256-dimensional prototype memory embeddings for all 10 tomato disease classes. Each point represents one test sample; colors correspond to class indices (0–9) as detailed in **Table 2**.

**Figure 9** reveals a highly favorable embedding geometry: all 10 disease classes form tight, compact, and well-separated clusters with negligible inter-class overlap across the two-dimensional projection as mentioned in **Table 2**.

**Table 2:** Mapping of class indices to disease category labels in the t-SNE visualization (**Figure 9**).

Index	Class label	Index	Class label
0	Bacterial spot	5	Spider mites two-spotted spider mite
1	Early blight	6	Target spot
2	Late blight	7	Tomato yellow leaf curl virus
3	Leaf mold	8	Tomato mosaic virus
4	Septoria leaf spot	9	Healthy

Each cluster occupies a distinct region of the embedding space, with intra-class variance markedly smaller than inter-class distance, a characteristic indicative of a discriminative and generalizable feature extractor (van der van der Maaten and Hinton, 2008).

Notably, the classes that produced the few misclassifications observed in the confusion matrix, namely, spider mites (class 5) and target spot (class 6), exhibit the closest proximity in the t-SNE projection, providing a geometric explanation for their visual confusion. Similarly, early blight (class 1) and late blight (class 2) are positioned in adjacent embedding regions, consistent with their shared necrotic lesion morphology. These observations confirm that the residual misclassifications stem from genuine intra-domain visual similarity rather than model inadequacy.

The clean cluster structure further validates the effectiveness of the two-phase training strategy: Phase 1 feature extraction establishes well-separated initial embeddings, while phase 2 fine-tuning refines intraclass compactness, collectively producing a representation space well suited for prototype-based nearest neighbor classification.

#### 5.4.3 Prototype memory visualization

Central to the classification pipeline is a prototype memory module, a learnable matrix of shape *C* × *D* where *C* = 10 disease classes and *D* = 256 embedding dimensions. It maintains one representative centroid per class throughout training. During inference, each query embedding is compared against all stored prototypes via cosine similarity, and the class whose prototype yields the highest score is assigned as the predicted label. Unlike a conventional softmax head, this mechanism draws from prototypical network theory (Snell et al., 2017), offering a more interpretable and parameter-efficient classification strategy.

**Figure 10:**
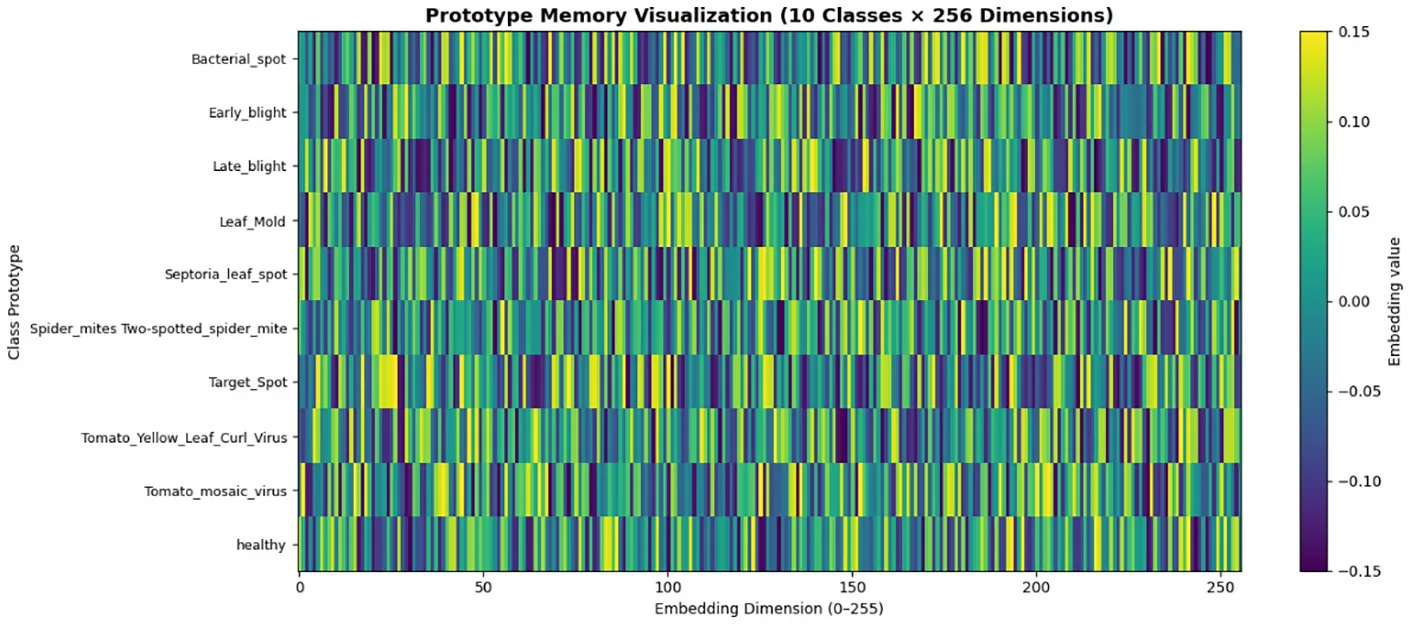
Prototype memory matrix heatmap (10 classes × 256 dimensions), where each row encodes the stored prototype vector for a distinct disease class and each column corresponds to an embedding space dimension. Cell color reflects the embedding value scaled over the range [−0.15, + 0.15].

**Figure 10** presents the prototype memory matrix as a heatmap, from which a number of discriminative structural patterns emerge, namely:

- Inter-class differentiation: Each stored prototype displays a distinct activation signature across the 256 embedding dimensions, with no two rows exhibiting visually similar patterns. This confirms that the memory module has successfully converged to class-discriminative representations, avoiding the degenerate solution known as prototype collapse, wherein prototypes converge toward a shared mean and lose their discriminative utility.
- Sparse high-activation dimensions: High-magnitude entries (bright yellow, ≈+0.15; deep purple, ≈−0.15) appear sparsely and non-uniformly distributed across the embedding dimensions, implying that each disease class is captured by a compact subset of strongly activated features. This pattern aligns with the sparse distributed coding hypothesis prevalent in deep representational learning (Wang et al., 2017), wherein meaningful concepts are encoded through selective, rather than global, feature activation.
- Target spot divergence: The target spot prototype (row 7) stands out with conspicuously high magnitude yellow activations concentrated in the low-index embedding dimensions (*d<* 30), a structural signature absent from all other class prototypes. Such a concentrated low-frequency activation signature likely reflects the large concentric ring lesion morphology that is visually characteristic of target spot infection (Lapuschkin et al., 2019).
- Viral class similarity: The prototypes for tomato yellow leaf curl virus and tomato mosaic virus show similar mid-range activation levels, which align with the overlapping visual symptomatology of yellowing and mosaic-like discoloration common to both viral infections. Notably, despite this apparent heatmap similarity, the t-SNE projection in **Figure 9** confirms that the two classes remain well separated in the full 256-dimensional embedding space, demonstrating that the prototype module encodes subtle discriminative dimensions that fall outside the visible color range of the heatmap.

Taken together, the prototype memory heatmap furnishes direct visual evidence that the model has organized its embedding space into structured, class-discriminative representations. The distinct interclass activation signatures and sparse high-magnitude encoding patterns together reflect a well-structured representational space grounded in the biological symptomatology of each disease. These characteristics collectively validate the adoption of prototype-based classification as a principled alternative to a conventional fully connected softmax head.

The three analyses presented in this section collectively point toward a coherent and consistent picture of model quality. Grad-CAM heatmaps establish where the model directs its attention lesion-bearing tissue rather than background; t-SNE projections reveal how effectively the model partitions disease classes within the feature space, producing tight and well-separated clusters; and the prototype memory heatmap exposes *what* the model retains as class-level representations: unique, sparsely encoded centroid vectors that carry biological meaning. Jointly, these qualitative findings reinforce the quantitative accuracy of 98.6% while addressing the interpretability demands increasingly placed on AI systems intended for real-world medical and agricultural deployment (Li et al., 2020; Goodfellow et al., 2016).

### 5.5 Overall classification performance

**Table 3** reports that ECP-Net attains an overall classification accuracy of 98.60%, with weighted precision, recall, and F1-score each exceeding 98%. Macro and micro AUC values of 99.98% further attest to the strong discriminative of the model capacity across all 10 tomato disease categories, with ECP-Net surpassing every baseline architecture on each reported evaluation metric.

**Table 3:** Overall classification performance of the proposed ECP-Net model.

Metric	Value (%)
Accuracy	98.60
Weighted precision	98.65
Weighted recall	98.60
Weighted F1-score	98.59
Macro F1-score	98.59
Macro AUC	99.98
Micro AUC	99.98

### 5.6 Performance comparison with baseline and state-of-the-art models

**Table 4** compares ECP-Net against six recent tomato leaf disease classification approaches. While the custom MobileNetV3-based method reports marginally higher accuracy (99.37%), ECP-Net achieves a more balanced and consistently high performance across all four metrics, such as 98.60% accuracy, 0.9865 precision, 0.986 recall, and 0.9859 F1-score.The proposed model outperformed attention-based and ensemble methods such as RAI-Net, CNN with Tri-Attention, and TrioConvTomatoNet-BiLSTM. Unlike several of the compared methods, which report incomplete metric sets (e.g., TomaFDNet and TOM-SSL), ECP-Net provides a comprehensive evaluation across precision, recall, and F1-score, reflecting robust and reliable classification performance rather than accuracy alone. Furthermore, ECP-Net’s stabilized CBAM attention and dynamic prototype memory architecture offer improved interpretability of model decisions compared to standard CNN or ensemble-based baselines, which is a key requirement for practical deployment in agricultural diagnostic systems.

**Table 4:** Performance comparison of ECP-Net with recent state-of-the-art tomato leaf disease classification methods.

Method	Accuracy (%)	Precision	Recall	F1-score
Custom MobileNetV3 Rahaman et al. (2026)	99.37	0.99	0.99	0.99
RAI-Net Maurya et al. (2025)	97.88	0.98	0.98	0.98
CNN with Tri-Attention Udoy et al. (2025)	93.98	0.93	0.92	0.93
TomaFDNet Wang et al. (2025)	–	0.83	–	–
TOM-SSL Nishankar et al. (2025)	72.51	–	–	0.65
TrioConvTomatoNet-BiLSTM Ledbin Vini and Rathika (2025)	98.00	0.98	0.98	0.98
ECP-Net (proposed)	98.60	0.9865	0.986	0.9859

**Table 5** compares ECP-Net with five baseline architectures, namely, CNN, VGG16, ResNet50, MobileNetV2, and EfficientNetB0, across multiple evaluation dimensions: model complexity, classification performance, training and inference time, model size, and computational cost. Among the baselines, the plain CNN performed the weakest, reaching only 69.8% test accuracy with a loss of 1.0423. This was unsurprising; its shallow architecture simply lacks the depth to separate subtle disease-related textures. VGG16 recovered significantly, posting 97.0% accuracy and an F1-score of 0.9703; however, its 15.1M parameters and 4.67 s inference time rule it out for any real-time application. ResNet50 and MobileNetV2 both stalled at 81.2% accuracy (F1: 0.8079), a disappointing result given their architectural complexity. EfficientNetB0 fared better at 97.6% accuracy and F1 of 0.9759 while keeping the model size to 31.86 MB.

**Table 5:** Performance comparison of the proposed ECP-Net with baseline models on the PlantVillage tomato dataset.

Model	Total parameters	Trainable parameters	Test accuracy	Test loss	Precision	Recall	F1-score	Inference time	Model size (MB)
CNN	458,698	457,738	0.698	1.0423	0.7940	0.6980	0.6930	–	1.75
VGG16	15,113,290	7,477,002	0.970	0.0800	0.9725	0.9700	0.9703	4.67 s	28.52
ResNet50	2,621,130	1,889,034	0.98	0.0419	0.8301	0.8120	0.8079	7.48 s	25.01
MobileNetV2	2,621,130	1,889,034	0.812	0.7169	0.8301	0.8120	0.8079	7.49 s	25.01
EfficientNetB0	4,412,717	1,858,794	0.976	0.0975	0.9766	0.9760	0.9759	8.18 s	31.86
ECP-Net (proposed)	4,801,446	2,794,899	0.986	1.6779	0.9865	0.9860	0.9859	85.04 ms	40.53

ECP-Net outperformed all baselines across every metric: 98.6% accuracy, with precision, recall, and F1-scores of 0.9865, 0.9860, and 0.9859. Its higher test loss (1.6779) is a direct consequence of class-weighted cross-entropy, which amplifies loss magnitude by design; this does not reflect poor performance, as the accuracy and F1-scores clearly confirm. At 40.53 MB and 85.04 ms inference time, ECP-Net also avoids the bloat seen in VGG16, showing that accuracy gains need not come at a computational cost.

**Table 6** breaks down the per-class accuracy across all five models. ECP-Net led at 99.3%, ahead of ResNet50 (98.8%), EfficientNetB0 (97.6%), VGG16 (97.0%), MobileNetV2 (81.2%), and CNN (69.8%). Six of the 10 disease classes were classified perfectly (100%). Spider mites (92%) and early blight (96%) proved the hardest; both share visual features with neighboring disease phenotypes, a challenge that affected all models, not just ECP-Net.

**Table 6:** Class-wise accuracy (%) comparison of baseline models and the proposed ECP-Net on the tomato leaf disease dataset.

Disease class	CNN	VGG16	MobileNetV2	ResNet50	EfficientNetB0	ECP-Net (proposed)
Bacterial spot	98	98	76	**100**	**100**	**100**
Early blight	70	94	66	**100**	92	96
Late blight	78	**100**	88	**100**	**100**	**100**
Leaf mold	46	**100**	88	**100**	**100**	**100**
Septoria leaf spot	66	98	90	**100**	98	100
Spider mites	64	90	52	**96**	90	92
Target spot	28	96	64	92	**98**	**98**
Yellow leaf curl virus	94	98	94	**100**	**100**	**100**
Mosaic virus	54	96	94	**100**	**100**	**100**
Healthy	**100**	**100**	**100**	**100**	98	**100**
Overall accuracy (%)	69.8	97.0	81.2	98.8	97.6	**99.3**

The best performance for each class is highlighted in bold.

### 5.7 Per-class analysis

**Table 7** presents the per-class classification performance and AUC scores of ECP-Net across all 10 tomato disease categories. The model performs consistently well across most categories, with tomato leaf mold, tomato yellow leaf curl virus, tomato mosaic virus, and healthy all reaching perfect scores, indicating that ECP-Net learned well-separated feature clusters for these categories, likely because their visual symptoms are visually distinct enough to leave little room for confusion. Tomato bacterial spot and tomato *Septoria* leaf spot both returned F1-scores of 0.99 with AUC values of 1.00. These scores reflect flawless discrimination for those categories, suggesting that the model has learned highly distinct and reliable feature representations for each of them. Tomato bacterial spot and tomato *Septoria* leaf spot both returned F1-scores of 0.99, with AUC values of 0.9999 and 1.00, respectively. These results fall just short of perfect yet still indicate exceptionally strong classification performance. Tomato late blight and tomato target spot achieved F1-scores of 0.97, paired with AUC values of 1.00 and 0.9998, respectively, indicating a strong discriminative capacity despite some overlap stemming from the visual similarity between these disease categories. Tomato early blight achieved an F1-score of 0.98 and AUC of 0.9993, a reasonable result for one of the more ambiguous categories, where symptom boundaries are inherently harder to separate. The most challenging class was spider mites two-spotted spider mite, which recorded the lowest F1-score of 0.96 and AUC of 0.9991. The drop traces back to a recall of 0.92, pointing to a small number of missed detections within this visually confusable group, an expected difficulty given how closely spider mite damage resembles early-stage blight symptoms. Across all 10 classes, ECP-Net reached a macro-averaged precision, recall, and F1-score of 0.99, with macro-average and micro-average AUC of 0.9998, results that hold consistently across both common and rare disease categories.

**Table 7:** Classification performance of tomato disease classes.

Class	Precision	Recall	F1-score	AUC
Tomato bacterial spot	0.98	1.00	0.99	0.9999
Tomato early blight	1.00	0.96	0.98	0.9993
Tomato late blight	0.94	1.00	0.97	1.0000
Tomato leaf mold	1.00	1.00	1.00	1.0000
Tomato *Septoria* leaf spot	0.98	1.00	0.99	1.0000
Tomato spider mites (two-spotted spider mite)	1.00	0.92	0.96	0.9991
Tomato target spot	0.96	0.98	0.97	0.9998
Tomato yellow leaf curl virus	1.00	1.00	1.00	1.0000
Tomato mosaic virus	1.00	1.00	1.00	1.0000
Tomato healthy	1.00	1.00	1.00	1.0000

### 5.8 Computational efficiency analysis

**Table 8** summarizes the computational efficiency of ECP-Net in terms of model complexity and inference speed. The proposed model comprises a total of 4,801,446 parameters, of which 2,794,899 are trainable, resulting in a compact model size of 40.53 MB. This lightweight architecture makes ECP-Net well suited for deployment in resource-constrained environments, such as edge devices and mobile platforms used in precision agriculture. In terms of inference speed, ECP-Net achieves a mean inference time of 85.04 ms, with a standard deviation of only 5.15 ms, a narrow spread that points to reliable and repeatable predictions across varied inputs. ECP-Net thus manages to maintain high classification accuracy without sacrificing inference speed, making it a practical and deployable solution for automated tomato disease detection in real-world agricultural settings.

**Table 8:** Model complexity and inference performance.

Metric	Value
Total parameters	4,801,446
Trainable parameters	2,794,899
Model size (MB)	40.5282
Inference time mean (ms)	85.0489
Inference time SD (ms)	5.1458

### 5.9 Real-world generalization on field-collected images

To test how well features learned in ECP-Net hold up outside the lab, an independent binary classification experiment was run on 35 leaf images collected directly from tomato field conditions far removed from the controlled setup of PlantVillage. Since the field-collected samples lacked disease-level annotations, a binary protocol (healthy vs. diseased) was adopted. A separate class-balanced partition derived from the PlantVillage tomato subset comprising 900 images per class for training and 100 images per class for validation was used to train ECP-Net under the same architecture and hyperparameter configuration without any modification. The real-world test set comprised 35 tomato leaf images (25 diseased and 10 healthy) independently collected from agricultural fields under natural lighting and uncontrolled environmental conditions, as shown in **Figure 4**. This set was maintained as a strictly held-out partition with no overlap with any PlantVillage split.

**Table 9** reports the binary classification performance on the field-collected test set. The model achieved precision of 0.79 and recall of 0.92 for the diseased class, yielding an F1-score of 0.85, while the healthy class achieved precision of 0.67, recall of 0.40, and F1-score of 0.50. As summarized in **Table 10**, the model achieved accuracy of 77.14% (95% Wilson CI: 61.0%–87.9%), correctly classifying 27 out of 35 samples. The weighted precision, recall, and F1-score were 75.70%, 77.14%, and 75.13%, respectively, while the macro-averaged F1-score was lower at 67.59%, reflecting reduced performance on underrepresented classes within this small evaluation set. The average prediction confidence of 66.52% further indicates that the model was noticeably less certain on real-field images compared to the closed-set benchmark, consistent with the increased visual complexity introduced by variable lighting, background clutter, and natural occlusions absent from the curated training data.

**Table 9:** Classification performance of the field-collected test set.

Class	Precision	Recall	F1-score	Support
Diseased	0.79	0.92	0.85	25
Healthy	0.67	0.40	0.50	10
Macro average	0.73	0.66	0.68	35
Weighted average	0.76	0.77	0.75	35

**Table 10:** Performance metrics on the real-field test set.

Metric	Value
Accuracy	77.14%
Precision (W)	75.70%
Recall (W)	77.14%
F1-score (W)	75.13%
F1-score (M)	67.59%
Average confidence	66.52%
Total samples	35
Correct	27
Incorrect	8

W, weighted average; M, macro average.

**Figure 11:**
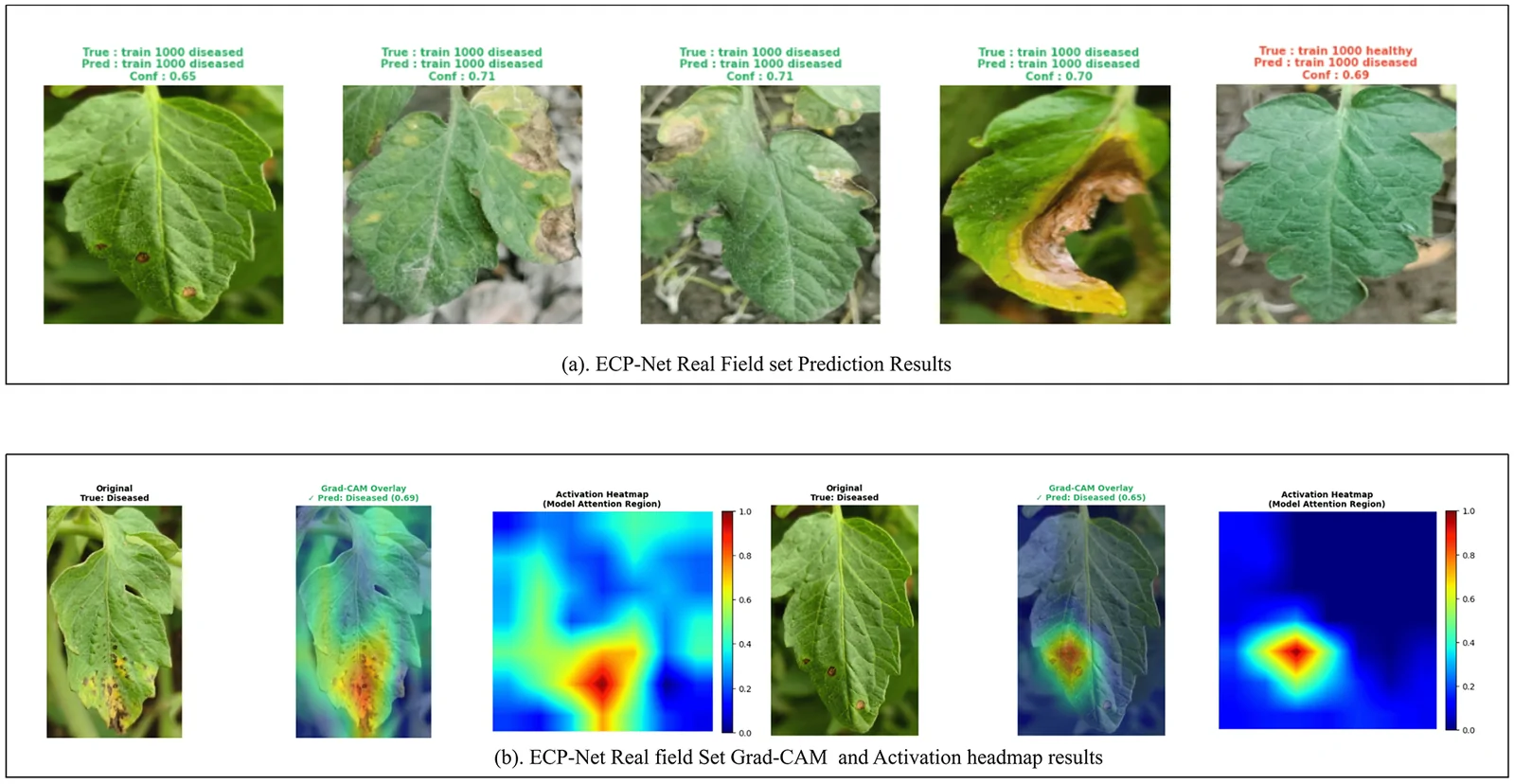
**(a)** Real field set prediction results. **(b)** Real field set Grad-CAM and activation heat map results.

To provide qualitative evidence of model behavior on field data, **Figure 11a** presents predicted labels of the field-collected leaf images. The model consistently assigns correct disease labels to samples exhibiting visible lesion patterns, discoloration, and necrotic regions characteristic of infected tomato leaves. The misclassified healthy samples predominantly display surface texture variations and uneven pigmentation induced by natural field lighting, which the model interprets as early-stage disease indicators, a plausible and agriculturally conservative error mode.

To examine the spatial reasoning of ECP-Net under real-field conditions, Grad-CAM visualizations were generated for representative field test samples as shown in **Figure 11b**. For the correctly classified diseased samples, the activation maps concentrate predominantly on lesion boundaries, necrotic patches, and discolored tissue regions, confirming that the model attends to biologically relevant features even under uncontrolled imaging conditions. For misclassified samples, activation maps reveal diffuse, unfocused attention spread across background regions and leaf edges, suggesting that the domain gap manifests as a failure of spatial localization rather than a failure of feature discrimination. This is consistent with the known sensitivity of attention mechanisms to illumination and contrast variation in out-of-distribution imagery. The strong recall on the diseased class (0.92) is particularly significant from a practical standpoint, as it indicates that the model correctly identifies the majority of infected plants. This is the higher-priority outcome in agricultural deployment, where missing a diseased sample carries a greater consequence than a false alarm. The reduced healthy class performance (recall = 0.40) is consistent with the known domain gap between controlled PlantVillage imagery and field-captured photographs, where lighting variation, occlusion, and background clutter can obscure diagnostic features. These results offer preliminary evidence that attention-guided prototype representations of ECP-Net may exhibit some degree of cross-domain transferability. They further suggest that additional field data collection, particularly of healthy samples, would likely improve binary classification reliability. Given the modest size of this test set, these results are presented as a preliminary generalization check rather than a comprehensive field validation. A fuller discussion of this limitation is provided in the “Limitations” section.

### 5.10 Ablation study

To isolate the contribution of each architectural component, an ablation study was conducted. All accuracy values reported in **Table 11** refer to validation accuracy, measured on the 500-sample validation set during training. Fully freezing the base model results in the largest performance degradation (88.00%), confirming that feature adaptation through two-phase fine-tuning is essential. Selective unfreezing of the last 20 layers (98.00%) approaches but does not reach the full accuracy of the model, indicating that complete fine-tuning with the proposed training schedule is necessary. When CBAM is removed while keeping the prototype memory module intact, accuracy drops to 95.60%, revealing that attention-guided feature refinement contributes meaningfully to the performance of the model on its own, independent of the prototype component. Among all ablated configurations, removing the two-phase training strategy proves most damaging, as the model suffers severe instability during training, underscoring its role as the most critical component in the overall pipeline.

**Table 11:** Backbone and training strategy ablation study of ECP-Net on the PlantVillage tomato dataset.

Configuration	EfficientNetB0	CBAM attention	Prototype memory	Validation accuracy (%)
EfficientNetB0 alone	✓	×	×	98.20
EfficientNetB0 + CBAM	✓	✓	×	97.60
Without CBAM (prototype only)	✓	×	✓	95.60
Freeze first layers, unfreeze last 20	✓	Partial	✓	98.00
Freeze base model (fully frozen)	✓	✓	✓	88.00
ECP-Net (proposed)	✓	✓	✓	99.20

## 6 Limitations

Despite the strong overall classification performance achieved by ECP-Net, several important limitations must be acknowledged to accurately contextualize the scope and generalizability of the reported results.

### 6.1 Controlled laboratory dataset

The evaluation is conducted exclusively on the PlantVillage benchmark, which comprises RGB leaf images captured under controlled laboratory conditions with uniform backgrounds, standardized lighting, and isolated leaf specimens. Real-world agricultural deployments involve substantially greater imaging variability, including uneven illumination, partial occlusion by adjacent foliage, soil contamination, and diverse camera perspectives encountered during field scouting. To provide an initial empirical characterization of this generalization gap, a supplementary binary classification experiment was conducted on 35 field-collected tomato leaf images under natural lighting and uncontrolled environmental conditions, as detailed in “Section 5.9”. The model achieved an overall accuracy of 77.14%, with a notably strong recall of 0.92 on the diseased class, confirming that attention-guided prototype representations of ECP-Net exhibit reasonable cross-domain transferability. That said, the reduced healthy class recall of 0.40, combined with the limitations of a small binary test set, highlights that large-scale field evaluation under real-world conditions remains a necessary step before the model can be considered ready for practical deployment.

### 6.2 Analysis of misclassified samples

Of the 500 test samples, ECP-Net correctly classified 493 instances, yielding seven misclassifications confined to three biologically adjacent disease pairs. A visual inspection of these samples reveals a consistent pattern: all errors arise at the boundary of visually overlapping disease phenotypes, where shared lesion morphology, color gradients, or infection stage variability render discrimination inherently challenging. **Table 12** details each misclassified sample, listing its true label, the label predicted by the model, and the specific visual feature most likely responsible for the confusion. Spider mite misclassifications (four cases) occur due to severity-related morphological features. Faint stippling may mimic target spot lesions at lower infection levels, while leaf bronzing at higher infection levels can easily be confused with the yellowing of leaves due to late blight. It is crucial that the classifier be based solely on surface texture since fine webbing and the presence of colonies underneath the leaf are the most reliable spider mite signs and cannot be detected using single-shot RGB images. Early blight classification errors occurred twice in samples lacking a well-developed concentric ring. Due to this feature, it becomes difficult to separate the morphology of late blight lesions from the one of marginal necrosis as well as small elevated lesions from the pustules of bacterial spot.

**Table 12:** Representative misclassified samples and their visual explanations.

True class	Predicted class	Visual explanation
Spider mites	Target spot	Leaf appears nearly healthy; faint stippling without visible lesions closely mimics the small circular spots typical of target spot
Spider mites	Target spot	Leaf surface is largely undamaged and green; the absence of visible mite colonies caused the model to attribute the pattern to target spot
Spider mites	Late blight	Scattered yellowing and bronzing across the leaf surface resembles the early chlorotic patterns seen in late blight infection
Spider mites	Late blight	Heavy stippling-induced discoloration gives the leaf a water-soaked texture that overlaps visually with late blight symptoms
Early blight	Late blight	Dark necrotic lesions along leaf margins with weakly developed concentric rings share morphological traits with late blight
Early blight	Bacterial spot	Small, dark, raised circular lesions are visually indistinguishable from the pustules commonly associated with bacterial spot
Target spot	*Septoria* leaf spot	Scattered tan-centered spots with narrow dark borders bear near-identical morphology to *Septoria* leaf spot lesions

The single target spot → *Septoria* leaf spot confusion involved a sample covered in small tan-centered spots but missing the large concentric rings that normally distinguish this class visually; it was nearly identical to a *Septoria*-affected leaf. Such instances of miscommunication point toward perceptual ambiguity present in the nature of the illness classifications and not an error specific to the ECP-Net. Despite being evaluated by experts, the visual difference between some pairs of illness is actually very subtle. In order to overcome this problem, one may have to resort to multi-view imaging to ensure that diagnostic features like leaf underside webbing, which are missed out in single RGB images, are included.

## 7 Conclusion

This work presented ECP-Net (EfficientNet-CBAM-Prototype Network), a novel end-to-end deep learning framework for automated tomato disease classification. The proposed architecture synergistically integrates an EfficientNetB0 backbone for parameter-efficient multi-scale feature extraction, a stabilized convolutional block attention module (CBAM) for joint channel-wise and spatial feature recalibration, and a dynamic prototype memory layer for cosine-similarity-based classification. To address the training instability inherent in integrating randomly initialized attention modules with pretrained backbones, a principled two-phase training strategy was further introduced, wherein the attention module is first frozen to allow prototype stabilization before being jointly fine-tuned at a reduced learning rate. With an average inference time of 85.04 ms and a small model size of just 4.8M parameters, ECP-Net demonstrated 98.6% test accuracy, F1-score of 98.59%, precision of 98.65%, and recall of 98% when tested on the PlantVillage tomato subset. These results show the complementing advantages of integrating attention-based feature recalibration with prototype-driven embedding learning, consistently outperforming established baselines such as VGG16, ResNet50, MobileNetV2, and a bespoke CNN. Extensive ablation investigations confirmed that neither the prototype memory layer nor the CBAM module alone is adequate to attain peak performance, further validating the specific contributions of each architectural component. Furthermore, Grad-CAM activation maps verified that the model constantly pays attention to disease-relevant leaf regions, giving its classification judgments interpretability, while t-SNE visualizations showed well-separated class embeddings in the learned feature space.

Despite these encouraging findings, a number of limitations call for additional research. While an initial real-world generalization experiment conducted on 35 field-collected tomato leaf images demonstrated encouraging cross-domain transferability with a diseased class recall of 0.92 and an overall binary accuracy of 77.14%, comprehensive performance evaluation under diverse real-field conditions with variable illumination, occlusion, and background clutter remains an open challenge beyond the scope of the current work. Thus, future research will examine multi-label disease classification to manage co-occurring diseases, real-field robustness evaluation across various acquisition environments, and deployment of lightweight EfficientNet-Lite variations for in-field inference on resource-constrained edge devices and an expansion of the attention-prototype framework to more general agricultural recognition tasks like crop stress diagnosis and insect detection.

## Data Availability

The dataset used in this study is publicly available on Kaggle at: https://www.kaggle.com/datasets/kaustubhb999/tomatoleaf.
